# A Mendelian randomization analysis of the causal effect of body mass index on biomarkers and renal failure risk

**DOI:** 10.1097/MD.0000000000051095

**Published:** 2026-10-30

**Authors:** Xin Liu, Wenwen Yang

**Affiliations:** a Department of Intensive Care Medicine, The First Hospital of Lanzhou University, Lanzhou, Gansu Province, China; b The First Clinical Medical College, Lanzhou University, Lanzhou, Gansu Province, China.

**Keywords:** Biomarkers, blood pressure, Body Mass Index, cystatin C, Mendelian Randomization, Renal Failure

## Abstract

Renal failure significantly impairs the kidneys’ ability to filter waste and maintain fluid balance. This study employed Mendelian Randomization (MR) to investigate the causal effect of BMI on 44 biomarkers related to renal failure. The genetic data were sourced from publicly available genome-wide association studies (GWAS) and biobank datasets, including blood cells, urinary electrolytes, enzymes, liver markers, lipids, and blood pressure. Single-nucleotide polymorphisms (SNPs) were used as instrumental variables (IVs) to assess the causal relationships and to identify significant mediators of the BMI-renal failure pathway. The study found that BMI is a risk factor for renal failure (OR = 1.324, *P* = .00185). Out of 44 biomarkers, nine were associated with renal failure, and five acted as mediators in the relationship between BMI and renal failure: cystatin C (12.34%), glucose (7.02%), glycated hemoglobin (HbA1c) (6.83%), diastolic blood pressure (6.37%), and systolic blood pressure (7.14%). The findings suggest that reducing BMI could improve renal health by addressing metabolic factors like blood glucose and blood pressure. This study highlights the causal pathways linking BMI to renal failure, underscoring the importance of weight management and metabolic control for prevention.

## 1. Introduction

Renal failure, a severe dysfunction of the kidneys, results in an inability to properly filter waste and maintain fluid balance, significantly affecting patient health.^[1–3]^ Obesity, diabetes, and hypertension are major risk factors for renal failure, with body mass index (BMI) playing a critical role in its progression.^[4–6]^ However, the mechanisms by which BMI affects renal function, as well as the specific roles of related biomarkers, are not yet fully understood. Mendelian Randomization (MR)^[7,8]^ provides a valuable framework to infer causal relationships between exposure (such as BMI) and outcomes (like biomarkers or disease states) by using genetic variants as instrumental variables (IVs). This study utilizes the MR approach to explore the causal effect of BMI on a broad range of biomarkers, including blood cells, urinary sodium, urinary potassium, urinary creatinine enzymatic, albumin, calcium levels, liver enzyme levels, apolipoprotein levels, bilirubin levels, cholesterol levels, C-reactive protein levels, phosphate levels, blood pressure, and blood glucose. It also assesses how these biomarkers may mediate the risk of renal failure. The aim is to uncover potential pathways by which BMI impacts kidney health and to identify key mediators in this relationship. The study focuses on the following questions – What is the direct impact of BMI on renal failure? How does BMI influence specific biomarkers that may mediate renal function? Which biomarkers are significantly associated with renal failure? What is the contribution of these biomarkers as mediators in the overall effect of BMI on renal failure? By addressing these questions, this research aims to clarify the causal pathways linking BMI, biomarkers, and renal failure, contributing to a deeper understanding of how interventions targeting BMI and its related metabolic pathways could improve renal health outcomes. This work is particularly relevant given the urgency of identifying effective prevention strategies for renal failure.

## 2. Methods

The analytical procedure of this study, as shown in Figure 1, is divided into four main steps. First, the direct relationship between BMI and renal failure is examined. Second, the connection between BMI and 44 biomarkers, which may serve as potential mediators, is explored. Third, the impact of these biomarkers on renal failure is evaluated. Finally, significant mediators are identified, and their mediating effects are quantified in relation to the overall influence of BMI on renal failure. MR relies on three main assumptions: the genetic variants should affect the outcome only through the exposure (no pleiotropy), be independent of confounders affecting both exposure and outcome, and have a strong association with the exposure to serve as valid instruments.

**Figure 1.**
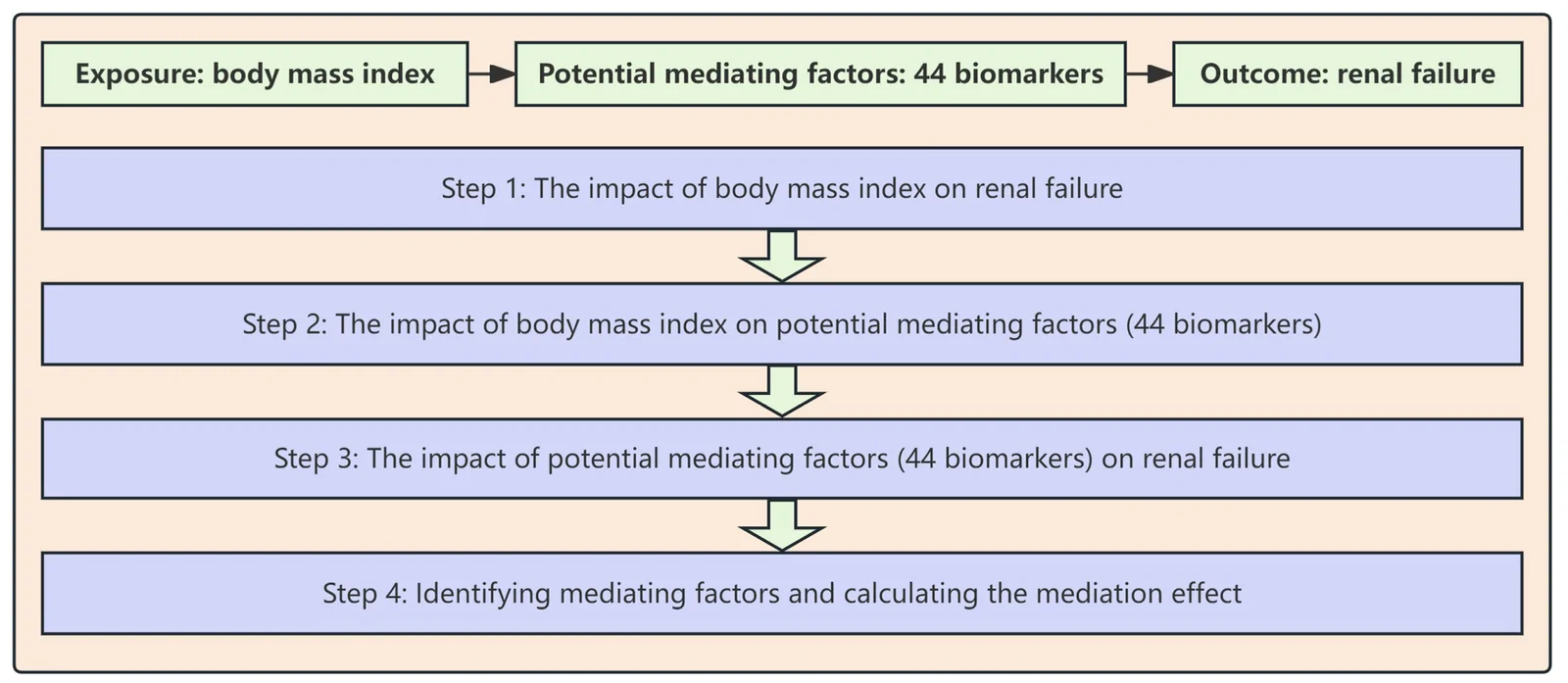
Analysis process flowchart. The analytical process of this study involves four steps, as illustrated in the figure. Step 1 examines the association between body mass index and renal failure. Step 2 investigates the influence of body mass index on potential mediators. Step 3 explores the relationship between potential mediators and renal failure. Step 4 calculates the ratio of the mediating effect to the total effect after identifying the mediating factors.

### 2.1. Data sources

Table 1 indicates that the GWAS data utilized for this analysis come from two sources: the IEU OpenGWAS project^[9]^ and the FinnGen Biobank.^[10]^ The BMI data were extracted by the IEU OpenGWAS project from a publicly published study.^[11]^ The 44 biomarkers were gathered from a publicly available study,^[12]^ whereas the renal failure data were derived from the FinnGen Biobank. These biomarkers primarily encompass categories such as urine markers, blood biochemistry, blood cells, blood pressure, and others. Additional information regarding the exposure, mediators, and outcomes is provided in Table 1. Approval from an ethics committee was not required for this research, as the MR analysis used publicly available datasets from open sources and did not involve any personal data.

**Table 1:** Information on exposure, mediating factors, and outcome datasets.

		Sample size	The article or biobank of the data source	Number of European-descent cases	Number of European-descent controls
exposure	Body mass index ‖ id:ieu-a-835	322,154	PMID:25673413	NA	NA
Potential mediating factors	White blood cell leukocyte count (UKB data field 30000) ‖ id:ebi-a-GCST90013976	396,621	PMID:34017140	NA	NA
	Red blood cell erythrocyte count (UKB data field 30010) ‖ id:ebi-a-GCST90013977	396,625	PMID:34017140	NA	NA
	Haemoglobin concentration (UKB data field 30020) ‖ id:ebi-a-GCST90013978	396,624	PMID:34017140	NA	NA
	Mean corpuscular volume (UKB data field 30040) ‖ id:ebi-a-GCST90013979	396,624	PMID:34017140	NA	NA
	Platelet count (UKB data field 30080) ‖ id:ebi-a-GCST90013980	396,621	PMID:34017140	NA	NA
	Mean platelet thrombocyte volume (UKB data field 30100) ‖ id:ebi-a-GCST90013981	396,616	PMID:34017140	NA	NA
	Lymphocyte count (UKB data field 30120) ‖ id:ebi-a-GCST90013982	395,949	PMID:34017140	NA	NA
	Monocyte count (UKB data field 30130) ‖ id:ebi-a-GCST90013983	395,949	PMID:34017140	NA	NA
	Neutrophill count (UKB data field 30140) ‖ id:ebi-a-GCST90013984	395,949	PMID:34017140	NA	NA
	Eosinophill count (UKB data field 30150) ‖ id:ebi-a-GCST90013985	395,949	PMID:34017140	NA	NA
	Basophil count ‖ id:ebi-a-GCST90013986	395,949	PMID:34017140	NA	NA
	Creatinine enzymatic in urine (UKB data field 30510) ‖ id:ebi-a-GCST90013987	396,837	PMID:34017140	NA	NA
	Potassium in urine (UKB data field 30520) ‖ id:ebi-a-GCST90013988	395,995	PMID:34017140	NA	NA
	Sodium in urine (UKB data field 30530) ‖ id:ebi-a-GCST90013989	396,020	PMID:34017140	NA	NA
	Albumin levels (UKB data field 30600) ‖ id:ebi-a-GCST90013990	357,968	PMID:34017140	NA	NA
	Alkaline phosphatase levels (UKB data field 30610) ‖ id:ebi-a-GCST90013991	389,883	PMID:34017140	NA	NA
	Alanine aminotransferase levels (UKB data field 30620) ‖ id:ebi-a-GCST90013992	389,733	PMID:34017140	NA	NA
	Apolipoprotein A levels (UKB data field 30630) ‖ id:ebi-a-GCST90013993	355,859	PMID:34017140	NA	NA
	Apolipoprotein B levels (UKB data field 30640) ‖ id:ebi-a-GCST90013994	388,022	PMID:34017140	NA	NA
	Peak expiratory flow (UKB data field 3064) ‖ id:ebi-a-GCST90013995	374,599	PMID:34017140	NA	NA
	Aspartate aminotransferase levels (UKB data field 30650) ‖ id:ebi-a-GCST90013996	388,490	PMID:34017140	NA	NA
	Direct bilirubin levels (UKB data field 30660) ‖ id:ebi-a-GCST90013997	332,739	PMID:34017140	NA	NA
	Urea levels (UKB data field 30670) ‖ id:ebi-a-GCST90013998	389,608	PMID:34017140	NA	NA
	Calcium levels (UKB data field 30680) ‖ id:ebi-a-GCST90013999	357,831	PMID:34017140	NA	NA
	Cholesterol levels (UKB data field 30690) ‖ id:ebi-a-GCST90014000	389,864	PMID:34017140	NA	NA
	Creatinine levels (UKB data field 30700) ‖ id:ebi-a-GCST90014001	389,678	PMID:34017140	NA	NA
	C reactive protein levels (UKB data field 30710) ‖ id:ebi-a-GCST90014002	389,057	PMID:34017140	NA	NA
	Cystatin C levels (UKB data field 30720) ‖ id:ebi-a-GCST90014003	389,834	PMID:34017140	NA	NA
	Gamma glutamyltransferase levels (UKB data field 30730) ‖ id:ebi-a-GCST90014004	389,672	PMID:34017140	NA	NA
	Glucose levels (UKB data field 30740) ‖ id:ebi-a-GCST90014005	357,580	PMID:34017140	NA	NA
	Glycated hemoglobin HbA1c levels (UKB data field 30750) ‖ id:ebi-a-GCST90014006	389,889	PMID:34017140	NA	NA
	High density lipoprotein cholesterol levels (UKB data field 30760) ‖ id:ebi-a-GCST90014007	357,810	PMID:34017140	NA	NA
	IGF 1 (UKB data field 30770) ‖ id:ebi-a-GCST90014008	387,834	PMID:34017140	NA	NA
	Direct low density lipoprotein levels (UKB data field 30780) ‖ id:ebi-a-GCST90014009	389,189	PMID:34017140	NA	NA
	Phosphate levels (UKB data field 30810) ‖ id:ebi-a-GCST90014010	357,302	PMID:34017140	NA	NA
	Sex hormone binding globulin levels (UKB data field 30830) ‖ id:ebi-a-GCST90014011	354,620	PMID:34017140	NA	NA
	Total bilirubin levels (UKB data field 30840) ‖ id:ebi-a-GCST90014012	388,303	PMID:34017140	NA	NA
	Testosterone levels (UKB data field 30850) ‖ id:ebi-a-GCST90014013	353,805	PMID:34017140	NA	NA
	Triglyceride levels (UKB data field 30870) ‖ id:ebi-a-GCST90014014	389,562	PMID:34017140	NA	NA
	Urate levels (UKB data field 30880) ‖ id:ebi-a-GCST90014015	389,404	PMID:34017140	NA	NA
	Vitamin D levels (UKB data field 30890) ‖ id:ebi-a-GCST90014016	373,045	PMID:34017140	NA	NA
	Diastolic blood pressure automated reading (UKB data field 4079) ‖ id:ebi-a-GCST90014017	385,801	PMID:34017140	NA	NA
	Systolic blood pressure automated reading (UKB data field 4080) ‖ id:ebi-a-GCST90014018	385,798	PMID:34017140	NA	NA
	Hand grip strength left (UKB data field 46) ‖ id:ebi-a-GCST90014019	406,552	PMID:34017140	NA	NA
outcome	Renal failure ‖ id:N14_RENFAIL	412,181	released in Round 10 by the FinnGen Biobank	15,475	396,706

GWAS = Genome-Wide Association Studies, IEU = Integrative Epidemiology Unit, NA = not applicable; More information about exposure, mediating factors,and outcomes is available at the IEU OpenGWAS project (https://gwas.mrcieu.ac.uk/) and the Finngen Biobank (https://r10.risteys.finngen.fi/).

### 2.2. Criteria for selecting IVs

Single-nucleotide polymorphisms (SNPs), which serve as IVs in Mendelian randomization, were identified according to specific criteria: a clumping window of 10,000 kb for physical proximity, a stringent linkage disequilibrium threshold (r^2^ < 0.001), and a genome-wide significance level (*P* < 5 × 10^−8^) to guarantee strong links with the exposure. To reduce bias and satisfy the assumptions of MR, SNPs that demonstrated a significant correlation with the outcome (*P* < 5 × 10^−8^) were removed from each analysis.

### 2.3. Statistical analysis

The primary approach applied in this research to detect causal relationships is the inverse-variance weighted (IVW) method, owing to its superior performance relative to the Weighted Median and MR-Egger methods. The IVW method’s effectiveness depends on having minimal or balanced horizontal pleiotropy.^[13]^ The MR-Egger method, allowing for a nonzero intercept, was employed to assess the occurrence of horizontal pleiotropy. To manage the rate of false positives in multiple hypothesis testing, adjustments were made using the False Discovery Rate (FDR) approach.^[14]^ The MR-PRESSO method was applied to identify outliers, and heterogeneity was evaluated using Cochran’s *Q*-test. All statistical analyses were conducted using R software (version 4.3.2) alongside the TwoSampleMR package (version 0.5.10).

### 2.4. Mediation analysis

To determine whether changes in biomarkers influenced by BMI affect the risk of renal failure, we conducted a two-step mediation MR analysis, treating 44 biomarkers as possible mediators. First, we examined the association between BMI and 44 biomarkers (β1). Next, we analyzed how these biomarkers are related to renal failure (β2). Biomarkers that showed associations with both BMI and renal failure were considered potential mediators. We then calculated their mediation effect (β1*β2) and determined their percentage contribution to the overall effect of BMI on renal failure.

## 3. Results

Our study initially analyzed the impact of BMI on renal failure, and the findings of this analysis are provided in Table S1, Supplemental Digital Content 1. We then examined the influence of BMI on 44 biomarkers, and the corresponding results can be found in Table S2, Supplemental Digital Content 2. Next, we assessed how the 44 biomarkers affect renal failure, and the related results are detailed in Table S3, Supplemental Digital Content 3. A total of 9 biomarkers associated with renal failure were identified based on the *P*-value, with the results displayed in Table 2. Table 3 outlines 5 biomarkers that were identified as significant mediators in the association between BMI and renal failure based on the *P*-value.

**Table 2:** The biomarkers associated with renal failure after removing outliers detected by the MR-PRESSO method, filtered based on the p-values obtained from the IVW method, with the exclusion of biomarkers exhibiting horizontal pleiotropy.

Serial Number	outcome	exposure	Number of SNPs	IVW_Beta	IVW_se	IVW_pval	IVW_q_value	egger_intercept	se	pval
1	Renal failure ‖ id:N14_RENFAIL	Urea levels (UKB data field 30670) ‖ id:ebi-a-GCST90013998	161	2.35E-01	5.92E-02	7.38E-05	6.49E-04	2.72E-03	3.37E-03	4.20E-01
2	Renal failure ‖ id:N14_RENFAIL	Creatinine levels (UKB data field 30700) ‖ id:ebi-a-GCST90014001	308	3.19E-01	4.96E-02	1.23E-10	5.22E-09	3.63E-03	2.17E-03	9.53E-02
3	Renal failure ‖ id:N14_RENFAIL	Cystatin C levels (UKB data field 30720) ‖ id:ebi-a-GCST90014003	315	2.69E-01	4.56E-02	3.59E-09	5.26E-08	3.29E-03	2.03E-03	1.06E-01
4	Renal failure ‖ id:N14_RENFAIL	Glucose levels (UKB data field 30740) ‖ id:ebi-a-GCST90014005	99	2.03E-01	6.65E-02	2.24E-03	9.87E-03	2.46E-03	3.47E-03	4.80E-01
5	Renal failure ‖ id:N14_RENFAIL	Glycated hemoglobin HbA1c levels (UKB data field 30750) ‖ id:ebi-a-GCST90014006	315	1.29E-01	3.55E-02	2.75E-04	2.01E-03	1.81E-03	1.77E-03	3.06E-01
6	Renal failure ‖ id:N14_RENFAIL	IGF 1 (UKB data field 30770) ‖ id:ebi-a-GCST90014008	343	−9.07E-02	3.36E-02	6.98E-03	2.79E-02	−3.19E-03	1.64E-03	5.28E-02
7	Renal failure ‖ id:N14_RENFAIL	Sex hormone binding globulin levels (UKB data field 30830) ‖ id:ebi-a-GCST90014011	265	−1.21E-01	3.70E-02	1.07E-03	5.87E-03	−2.76E-03	1.74E-03	1.14E-01
8	Renal failure ‖ id:N14_RENFAIL	Diastolic blood pressure automated reading (UKB data field 4079) ‖ id:ebi-a-GCST90014017	182	1.38E-01	6.56E-02	3.50E-02	1.28E-01	−1.16E-03	3.83E-03	7.63E-01
9	Renal failure ‖ id:N14_RENFAIL	Systolic blood pressure automated reading (UKB data field 4080) ‖ id:ebi-a-GCST90014018	217	3.67E-01	5.79E-02	2.37E-10	5.22E-09	9.38E-04	3.14E-03	7.65E-01

We identified biomarkers associated with renal failure after removing outliers detected by the MR-PRESSO method, filtered them based on the *P*-values obtained from the IVW method, and excluded those biomarkers exhibiting horizontal pleiotropy. The IVW method, known for its heightened sensitivity in detecting causality, serves as our primary tool to ascertain the presence of a causal relationship. The MR-Egger method is also employed for detecting horizontal pleiotropy due to its allowance for the presence of nonzero intercepts (The columns in the table corresponding to “egger_intercept,” “se,” and “pval”), a *P*_value below .05 suggests the existence of horizontal pleiotropy.

IVW = Inverse Variance Weighted method, MR_Egger = MR-Egger method, pval = *P*-value, q_value = The *P* value post FDR method (Benjamini and Hochberg) corrected, SNPs = Single-nucleotide polymorphisms.

**Table 3:** The mediators between body mass index and renal failure.

Serial Number	The impact of body mass index on mediators										The impact of mediators on renal failure										The mediation effect	The proportion of the mediation effect to the total effect
	outcome	exposure	Number of SNPs	IVW_Beta	IVW_se	IVW_pval	IVW_q_value	egger_intercept	se	pval	outcome	exposure	Number of SNPs	IVW_Beta	IVW_se	IVW_pval	IVW_q_value	egger_intercept	se	pval		
1	Cystatin C levels (UKB data field 30720) ‖ id:ebi-a-GCST90014003	Body mass index ‖ id:ieu-a-835	59	1.29E-01	2.02E-02	1.88E-10	1.03E-09	1.26E-03	1.64E-03	4.47E-01	Renal failure ‖ id:N14_RENFAIL	Cystatin C levels (UKB data field 30720) ‖ id:ebi-a-GCST90014003	315	2.69E-01	4.56E-02	3.59E-09	5.26E-08	3.29E-03	2.03E-03	1.06E-01	3.47E-02	12.34%
2	Glucose levels (UKB data field 30740) ‖ id:ebi-a-GCST90014005	Body mass index ‖ id:ieu-a-835	63	9.71E-02	1.93E-02	4.75E-07	1.61E-06	2.00E-03	1.50E-03	1.89E-01	Renal failure ‖ id:N14_RENFAIL	Glucose levels (UKB data field 30740) ‖ id:ebi-a-GCST90014005	99	2.03E-01	6.65E-02	2.24E-03	9.87E-03	2.46E-03	3.47E-03	4.80E-01	1.97E-02	7.02%
3	Glycated hemoglobin HbA1c levels (UKB data field 30750) ‖ id:ebi-a-GCST90014006	Body mass index ‖ id:ieu-a-835	60	1.49E-01	2.37E-02	3.71E-10	1.81E-09	1.47E-03	1.84E-03	4.27E-01	Renal failure ‖ id:N14_RENFAIL	Glycated hemoglobin HbA1c levels (UKB data field 30750) ‖ id:ebi-a-GCST90014006	315	1.29E-01	3.55E-02	2.75E-04	2.01E-03	1.81E-03	1.77E-03	3.06E-01	1.92E-02	6.83%
4	Diastolic blood pressure automated reading (UKB data field 4079) ‖ id:ebi-a-GCST90014017	Body mass index ‖ id:ieu-a-835	60	1.30E-01	2.44E-02	1.16E-07	4.64E-07	3.02E-03	1.94E-03	1.26E-01	Renal failure ‖ id:N14_RENFAIL	Diastolic blood pressure automated reading (UKB data field 4079) ‖ id:ebi-a-GCST90014017	182	1.38E-01	6.56E-02	3.50E-02	1.28E-01	-1.16E-03	3.83E-03	7.63E-01	1.79E-02	6.37%
5	Systolic blood pressure automated reading (UKB data field 4080) ‖ id:ebi-a-GCST90014018	Body mass index ‖ id:ieu-a-835	60	5.47E-02	2.27E-02	1.60E-02	2.35E-02	1.73E-03	1.81E-03	3.43E-01	Renal failure ‖ id:N14_RENFAIL	Systolic blood pressure automated reading (UKB data field 4080) ‖ id:ebi-a-GCST90014018	217	3.67E-01	5.79E-02	2.37E-10	5.22E-09	9.38E-04	3.14E-03	7.65E-01	2.01E-02	7.14%

Through our analysis, we have found that 5 biomarkers serve as mediators between body mass index and renal failure. The mediation effect is calculated as the product of the beta coefficient for the association between body mass index and mediators and the beta coefficient for the association between mediators and renal failure. The mediation effect refers to the effect of body mass index on renal failure mediated by these mediators, while the direct effect refers to the direct impact of body mass index on renal failure (total effect). The IVW method, known for its heightened sensitivity in detecting causality, serves as our primary tool to ascertain the presence of a causal relationship. The MR-Egger method is also employed for detecting horizontal pleiotropy due to its allowance for the presence of nonzero intercepts (the columns in the table corresponding to “egger_intercept,” “se,” and “pval”), a *P*-value below 0.05 suggests the existence of horizontal pleiotropy.

IVW = Inverse Variance Weighted method, MR_Egger = MR-Egger method, pval = *P*-value, q_value = The *P* value post FDR method (Benjamini and Hochberg) corrected, SNPs = Single-nucleotide polymorphisms.

### 3.1. The effect of BMI on renal failure

Our analysis revealed that BMI serves as a risk factor for renal failure (OR = 1.324, *P* = .00185). Additional details are available in Table S1, Supplemental Digital Content 1.

### 3.2. The impact of biomarkers on renal failure and the biomarkers mediating the relationship between body mass index and renal failure

Our analysis revealed that 9 out of the 44 biomarkers included in our study are associated with renal failure, specifically urea (Beta = 0.235, *P* < .0001), creatinine (Beta = 0.319, *P* < .0001), cystatin C (Beta = 0.269, *P* < .0001), glucose (Beta = 0.203, *P* = .00224), glycated hemoglobin HbA1c (Beta = 0.129, *P* = .000275), IGF 1 (Beta = -0.0907, *P* = .00698), sex hormone binding globulin (Beta = -0.121, *P* = .00107), diastolic blood pressure (Beta = 0.138, *P* = .035), and systolic blood pressure (Beta = 0.367, *P* < .0001). Further information can be found in Table 2 and Table S3, Supplemental Digital Content 3. By pinpointing biomarkers associated with both BMI and renal failure, we identified 5 biomarkers acting as mediators in this relationship, specifically cystatin C (12.34%), glucose (7.02%), glycated hemoglobin HbA1c (6.83%), diastolic blood pressure (6.37%), and systolic blood pressure (7.14%). Further information can be found in Table 3.

## 4. Discussion

This study used an MR approach to investigate the causal relationship between BMI, a range of biomarkers, and the risk of renal failure. The findings provide new insights into how increased BMI can impact renal health, mediated by specific biomarkers. Overall, our results strengthen the evidence that BMI is a significant risk factor for renal failure and highlight the importance of certain biomarkers that serve as mediators in this relationship. The MR analysis confirmed that elevated BMI is causally associated with an increased risk of renal failure (OR = 1.324, *P* = .00185). This aligns with the existing literature suggesting that obesity and higher BMI are strong predictors of chronic kidney disease and other renal complications.^[15–17]^ Our results reinforce the potential of targeting obesity as a modifiable factor in the prevention of renal failure. Through mediation analysis, we identified 29 potential biomarkers out of 44 that may be influenced by BMI. Among these 44 biomarkers, 9 were significantly associated with renal failure, with 5 acting as mediators between BMI and renal failure. The identified biomarkers, including cystatin C, glucose, glycated hemoglobin (HbA1c), diastolic blood pressure, and systolic blood pressure, support a multifactorial pathway by which BMI influences renal function. These biomarkers contributed varying effect sizes to the relationship between BMI and renal failure, with cystatin C having the largest mediating effect (12.34%). Glucose also played a notable role, contributing 7.02% to the mediation effect, highlighting its role in metabolic and renal health. Similarly, glycated hemoglobin HbA1c, diastolic blood pressure, and systolic blood pressure contributed 6.83%, 6.37%, and 7.14%, respectively. This provides a nuanced understanding of how multiple pathways link obesity and renal dysfunction, emphasizing that addressing metabolic factors such as blood glucose and blood pressure could mitigate renal failure risks in individuals with elevated BMI.

Obesity, diabetes, and hypertension are major risk factors for chronic kidney disease.^[4,5]^ Hypertension can lead to glomerulosclerosis, which subsequently causes a decline in renal function.^[18]^ Our study suggests that diabetes (as evidenced by glucose and HbA1c levels) and hypertension (as indicated by blood pressure levels) are important pathways linking BMI to renal outcomes. The strong mediating effect of cystatin C suggests that kidney function, specifically glomerular filtration, plays a pivotal role in this relationship. Given the complex interplay of these biomarkers, targeted interventions aimed at reducing BMI might benefit renal health through these pathways. A study^[19]^ found no association between C-reactive protein, leukocyte count, and rapid decline in renal function. Our study also found that blood cells (such as leukocyte count, erythrocyte count, platelet count, lymphocyte count, monocyte count, neutrophil count, eosinophil count, and basophil count), urinary sodium, urinary potassium, urinary creatinine enzymatic, albumin, calcium levels, liver enzyme levels, apolipoprotein levels, bilirubin levels, cholesterol levels, C-reactive protein levels, and phosphate levels were not associated with the risk of renal failure. By identifying the mediating role of diabetes and hypertension in the BMI-renal function relationship, our study highlights potential avenues for targeted interventions to mitigate kidney disease risk through weight management and blood pressure control. Additionally, the lack of association between a broad range of blood and urinary biomarkers and renal failure risk reinforces the need to explore other potential pathways and mechanisms. This research contributes to a better understanding of how modifiable risk factors like BMI can be targeted to improve renal health.

This study’s strength lies in its use of a robust MR framework to infer causal relationships, reducing potential biases associated with confounding and reverse causation.^[7]^ Additionally, the use of multiple statistical methods, including the inverse-variance weighted (IVW), MR-Egger, and MR-PRESSO, along with adjustment for multiple testing through the False Discovery Rate (FDR) approach, enhances the reliability of our findings. However, some limitations should be considered. While MR can provide evidence for causality, the use of genetic variants as instrumental variables assumes no pleiotropy, which, if violated, could bias the results. Although the MR-Egger and MR-PRESSO tests were employed to detect and account for horizontal pleiotropy and outliers, residual bias may remain. Additionally, the analysis was based on available genome-wide association study (GWAS) data, and future studies might benefit from larger sample sizes and more diverse populations to validate these findings further.

## 5. Conclusion

Our study identifies key biomarkers that mediate the relationship between BMI and renal failure. The mediators highlight critical pathways, particularly related to metabolic health and cardiovascular function, that could be targeted to prevent renal failure in individuals with obesity or high BMI. Further research into these biomarkers and their biological mechanisms may open new avenues for intervention and improve renal health outcomes in populations at risk.

## Acknowledgments

Special thanks to the UK Biobank, the FinnGen Biobank, and the IEU open GWAS project developed by The MRC Integrative Epidemiology Unit (IEU) at the University of Bristol.

## Author contributions

**Conceptualization:** Xin Liu, Wenwen Yang.

**Data curation:** Xin Liu, Wenwen Yang.

**Formal analysis:** Xin Liu, Wenwen Yang.

**Funding acquisition:** Xin Liu, Wenwen Yang.

**Investigation:** Xin Liu, Wenwen Yang.

**Methodology:** Xin Liu, Wenwen Yang.

**Visualization:** Xin Liu, Wenwen Yang.

**Writing – original draft:** Xin Liu, Wenwen Yang.

**Project administration:** Wenwen Yang.

**Resources:** Wenwen Yang.

**Software:** Wenwen Yang.

**Supervision:** Wenwen Yang.

**Validation:** Wenwen Yang.






